# Color visual acuity in preperimetric glaucoma and open-angle glaucoma

**DOI:** 10.1371/journal.pone.0215290

**Published:** 2019-04-17

**Authors:** Junko Ouchi, Hiroshi Kunikata, Kazuko Omodaka, Haruka Sato, Hiroyuki Sato, Azusa Ito, Naoko Aizawa, Yoshiki Tanaka, Kazuo Ichikawa, Toru Nakazawa

**Affiliations:** 1 Department of Ophthalmology, Tohoku University Graduate School of Medicine, Sendai, Japan; 2 Department of Retinal Disease Control, Tohoku University Graduate School of Medicine, Sendai, Japan; 3 Department of Ophthalmic Imaging and Information Analytics, Tohoku University Graduate School of Medicine, Sendai, Japan; 4 Chukyo Eye Clinic, Nagoya, Japan; 5 Department of Advanced Ophthalmic Medicine, Tohoku University Graduate School of Medicine, Sendai, Japan; Bascom Palmer Eye Institute, UNITED STATES

## Abstract

**Purpose:**

To investigate the clinical significance of color visual acuity (CVA) in preperimetric glaucoma (PPG) and open-angle glaucoma (OAG).

**Methods:**

A total of 123 eyes of 73 subjects (22 normal eyes, 14 PPG eyes, and 87 OAG eyes; mean age: 44.9 ± 10.1 years, age range: 21–64 years) were enrolled. CVA was tested for red, green-yellow, blue-green and blue-purple with a newly developed test.

**Results:**

There was no statistical difference in clinical background factors, including age, sex, intraocular pressure, or spherical equivalent between the three groups. Red VA and blue-green VA were significantly worse in the OAG eyes than in the normal eyes (*P* = 0.008 and *P* = 0.015, respectively), although green-yellow VA and blue-purple VA were not significantly worse. Furthermore, red VA and blue-green VA were significantly correlated with MD in a group of eyes with either PPG or OAG (r = -0.23, *P* = 0.023; r = -0.25, *P* = 0.012, respectively), but green-yellow VA and blue-purple VA were not.

**Conclusion:**

Red VA and blue-green VA were detectably worse in eyes with OAG, in close association with the degree of functional loss. This suggests that measuring CVA with the new color test described here may be a promising supplement to existing methods of detecting glaucoma and evaluating its severity.

## Introduction

Glaucoma affects over 70 million people worldwide and is the second most common cause of blindness.[1, 2] It is a group of diseases characterized by progressive, irreversible optic neuropathy and degeneration of the retinal ganglion cells and their axons, causing a corresponding loss of the visual field (VF). Thus, simple techniques for the early detection of glaucoma, as well as improved methods for preventing its progression, are important goals of public health research that would have a valuable social impact. Glaucomatous axonal loss and associated structural changes have long been known to precede the manifestation of VF defects, as measured with standard automated perimetry (SAP).[3] New advances in imaging methods, particularly optical coherence tomography (OCT), have allowed the early detection of structural changes, such as the loss of macular thickness and circumpapillary retinal nerve fiber layer thickness (cpRNFLT), enabling new research into preperimetric glaucoma (PPG).[4–11] However, the glaucomatous loss of cpRNFLT is difficult to measure in many eyes, because of various confounding factors, such as disc deformation, tilted disc, and peripapillary atrophy.[12, 13] Thus, other approaches are necessary to complement existing structural analyses and to improve the early detection of glaucoma.

Foveal function, including visual acuity (VA) and retinal sensitivity, is very important for quality of life. Even though glaucoma primarily affects the peripheral visual field, it can also affect foveal function. Previously, we found that cpRNFLT in the mid-temporal area was significantly correlated to VA in patients with glaucoma, and could accurately predict decreased VA.[14] However, even though VA decreases at an early stage in some glaucoma patients, in most cases it is preserved until the later stages.[15] This makes it difficult to detect early glaucoma with standard testing of central VA. Our recent work showed that functional visual acuity (FVA), a functional test to assess dynamic changes in central visual function, was impaired in early glaucoma, and that FVA was correlated with macular structure in the papillomacular bundle.[16] This suggests that FVA, an achromatic aspect of central visual function, may be a new, promising biomarker for the early detection of glaucoma. Additionally, the ratio between the resolution of chromatic and achromatic vision has been reported to be lower than normal at certain locations in certain individuals with early glaucoma.[17]

The human visual system is capable of processing chromatic (i.e., color) information, not only the achromatic information evaluated by conventional clinical test parameters, such as VA and contrast sensitivity. Furthermore, both the L- and M-cones are believed to be preferentially affected in experimental models of glaucoma, leading to acquired color vision loss. This suggests that the ability of the eye to sharply resolve objects of different colors, known as color visual acuity (CVA), might change with the progression of glaucoma, and that this impairment might differ with different colors. Recent research has established a method to measure CVA for the first time.[18, 19] This method uses a personal computer with a specially-calibrated liquid crystal display (LCD) with highly accurate color reproduction.[18, 19] Here, we developed a paper-based method of measuring CVA as a simpler alternative. We hypothesized that CVA might change for different colors in eyes with glaucoma or PPG, and that these changes might be related to glaucoma severity. To confirm this hypothesis, we investigated CVA in patients with PPG and open-angle glaucoma (OAG), in addition to healthy controls, using a paper-based test.

## Materials and methods

This was a single-center, cross-sectional study. This research followed the tenets of the Declaration of Helsinki and was approved by the Institutional Review Board of the Tohoku University Graduate School of Medicine. Written informed consent was obtained from all subjects.

### Subjects

Normal subjects, patients with PPG, and patients with glaucoma were recruited from Tohoku University Hospital. All subjects were at least 20 years old and had refractive error within the range of +3.00 to -6.00 diopters (including some patients with emmetropia), reliable performance in visual field testing (fixation errors < 20%, false positives < 33%, and false negatives < 33%), and no cataract progression. The inclusion criteria for the normal eyes were: best-corrected visual acuity (VA) ≥ 20/20; intraocular pressure (IOP) < 22 mmHg; normal findings in slit lamp and funduscopic examinations; and a visual field within the normal limits of the Anderson-Patella classification.[20] The inclusion criteria for the PPG eyes were: best-corrected VA ≥ 20/20; IOP < 22 mmHg without any treatment to lower IOP; a normal, open angle in a gonioscopic examination; glaucomatous changes in the optic disc, including neuroretinal rim thinning, notching and cupping; a visual field within the normal limits of a glaucoma hemifield test, with pattern standard deviation (PSD) greater than 5%, confirmed in at least two examinations. The inclusion criteria for the glaucoma, i.e., OAG, eyes were: best-corrected VA ≥ 20/20; glaucomatous changes in the optic disc; visual field defects conforming to the Anderson-Patella classification, confirmed in at least two visual field examinations; a normal, open angle in a gonioscopic examination; and IOP < 22 mmHg if any treatments to lower IOP were used. Ongoing medical treatment, including IOP-lowering topical medication in the patients with primary OAG, was not discontinued before the examination.

The exclusion criteria for all subjects were: best-corrected VA < 20/20; central visual field defects; clinical evidence or history of ocular disorders such as corneal, retinal and neural disease; presence of blood flow-affecting systemic disease requiring medical treatment; and IOP ≥ 21 mmHg.

### Measurement of clinical parameters

Subjects underwent an ophthalmological and general examination, which comprised the following: slit lamp and funduscopic examination, gonioscopy, IOP measurement with Goldmann applanation tonometry, spherical equivalent (SE) measurement with auto refractometry and mean deviation (MD) measurement with SAP using the Swedish Interactive Threshold Algorithm (Standard 24–2) of the Humphrey Field Analyzer (Carl Zeiss Meditec Inc., Dublin, California). Visual field measurements with fixation losses of more than 20%, or with more than 33% false positives or false negatives, were excluded from the analysis. MD was used for the evaluation of visual field function.

### Color visual acuity measurements

**Fig 1:**
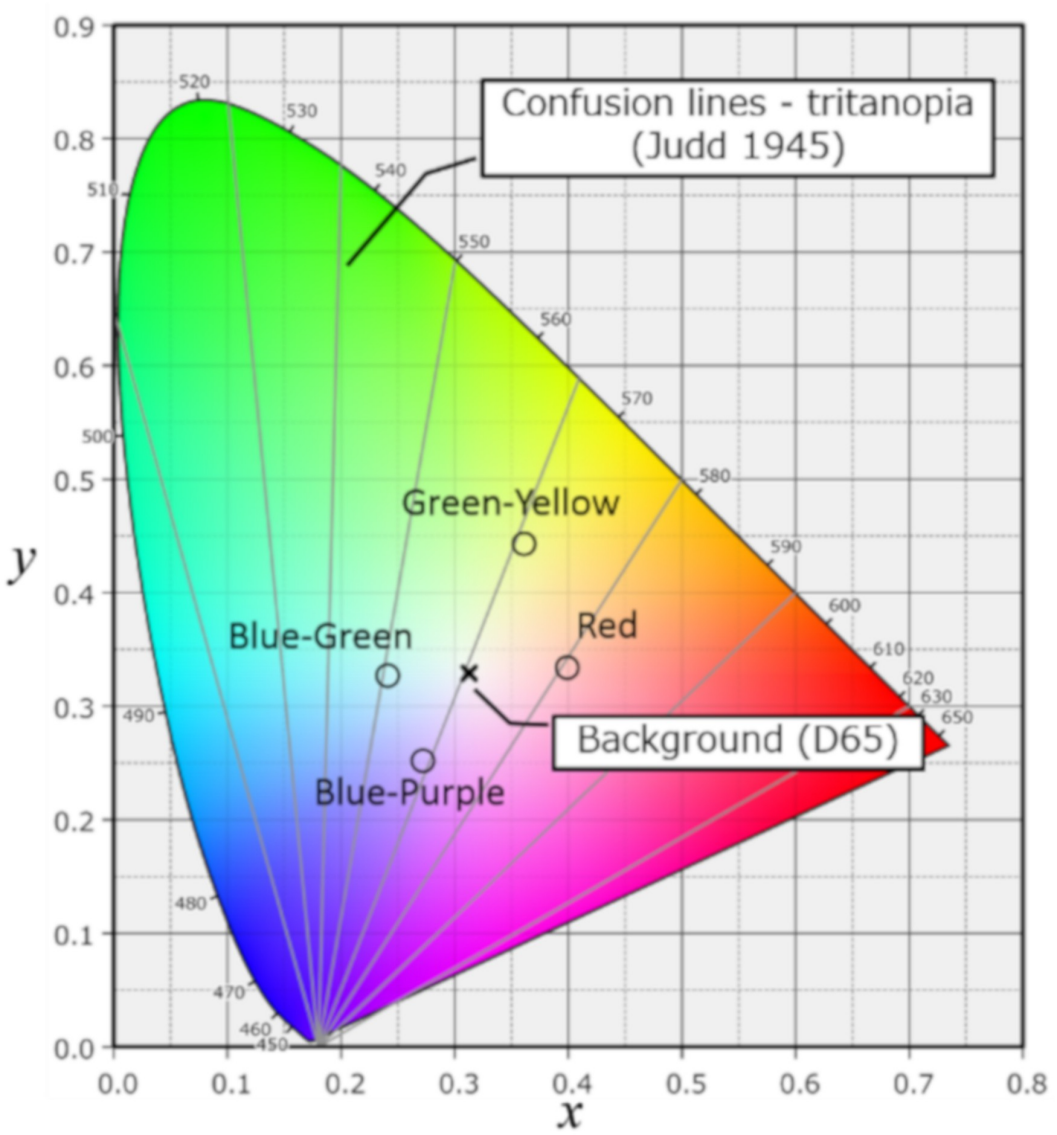
Chromaticity points of the four tested color stimuli and the background. We tested color visual acuity for red, green-yellow, blue-green and blue-purple using a paper-based chart with Landolt’s rings (test at 3 meters). The four colors are the same as those used in the New Color Test, a color arrangement test. The chromaticity points of each stimulus color and of the background (D65) are shown.

In order to easily evaluate color vision performance with CVA in a clinical setting, we developed a paper-based chart. This chart showed a colored Landolt’s ring against an achromatic (i.e., zero- saturation) background, which we carefully checked and calibrated for color accuracy. The luminance of the colored Landolt’s rings and the uncolored background were similar under various clinical lighting conditions during the initial design of the paper-based charts, as confirmed with a contact colorimeter. The chart was shown to the subjects at 3 meters after complete correction for refractive errors. We tested CVA for black-white (BW), red (R), green-yellow (GY), blue-green (BG) and blue-purple (BP). These four colors are all used in the New Color Test, a color arrangement test.[18] The sizes of the Landolt’s rings on the chart were as follows, expressed as the equivalent in logMAR units: R and BG: 1.05, 1.00, 0.82, 0.70, 0.52, 0.40, 0.30, 0.22, 0.15, 0.10, 0.05, 0, -0.08, -0.18 and -0.30; GY and BP: 1.10, 1.05, 1, 0.82, 0.70, 0.52, 0.40, 0.22 and 0.15. If had a subject had GY or BP CVA better than 0.7, they were asked to step back to 5m to simulate a smaller target. Decimal CVA was calculated line-by-line; success was defined as accurate identification of at least 3 of 5 rings in each row. The chromaticity of the colors, and of the uncolored background (D65) are shown in Fig 1. S1 Table shows characteristics of the measured chromaticity of the background (D65: (x, y) = (0.3127, 0.329)) and Landolt’s rings, the delta Y between them (i.e., Landolt’s ring—background), theoretical target chromaticity and Michelson contrast. These values were measured with the Konica-Minolta FD7, with D65 set as the reference light source. All tests were performed manually, not with an automatic program, in a room lit with uncalibrated lamps from a variety of manufacturers. D65 was chosen as the reference light source as the best compromise for the variety of light sources. Each measurement took about 5 minutes.

### Statistical analysis

The data are presented as the mean ± standard deviation or as percentages. The statistical analyses were performed with JMP software (Pro version 10.0.2, SAS Institute Japan Inc., Tokyo, Japan). The Wilcoxon/Kruskal-Wallis test (rank sums) with Bonferroni correction was used to determine the significance of differences between groups in age, IOP, SE, MD and CVA for different colors. Fisher’s test with Bonferroni correction was used to determine the significance of differences between groups in sex distribution. The Mann-Whitney U test with Bonferroni correction was used to determine the significance of differences between groups in CVA for different colors. The significance level was set at P < 0.017 in the Wilcoxon/Kruskal-Wallis test, Fisher’s test and Mann-Whitney U test. Finally, Spearman’s rank correlation test was used to evaluate single correlations between MD and CVA for each color, i.e., BW VA, R VA, GY VA, BG VA, and BP VA. The significance level was set at *P* < 0.05 in the Spearman’s rank correlation test.

## Results

A total of 123 eyes of 73 subjects (22 normal eyes, 14 PPG eyes, and 87 glaucoma eyes; mean age: 44.9 ± 10.1 years, age range: 21–64 years) were enrolled.

Table 1 shows the characteristics of the normal, PPG and glaucoma eyes. The three groups differed significantly in MD, but not in other clinical background factors, including age, sex, IOP, and SE.

**Table 1:** Characteristics of normal, preperimetric glaucoma and glaucoma eyes.

	Normal (N = 22)	PPG (N = 14)	Glaucoma (N = 87)	*P* value		
				Normal vs. PPG	PPG vs. glaucoma	Normal vs. glaucoma
Age (years)	41.4±12.2	45.0±11.5	45.8±9.2	0.33^a^	0.97^a^	0.09^a^
Sex (M:F)	11:11	5:9	35:52	0.40^b^	0.75^b^	0.41^b^
IOP (mmHg)	13.9±1.9	15.6±2.8	14.6±3.0	0.05^a^	0.22^a^	0.24^a^
SE (D)	-2.2±1.8	-4.4±3.2	-3.2±2.2	0.16^a^	0.37^a^	0.09^a^
MD (dB)	-0.2±0.9	0.8±1.0	-7.7±7.0	0.01^a^	<.001^a^	<.001^a^
CVA (logMAR)						
BW	-0.2±0.1	-0.2±0.1	-0.2±0.1	0.82^a^	0.88^a^	0.21^a^
R	0.1±0.1	0.2±0.2	0.2±0.2	0.71^a^	0.30^a^	0.008^a^
GY	0.4±0.1	0.4±0.2	0.5±0.2	0.73^a^	0.51^a^	0.25^a^
BG	0.1±0.2	0.2±0.1	0.3±0.3	0.82^a^	0.10^a^	0.015^a^
BP	0.7±0.2	0.8±0.2	0.8±0.3	0.40^a^	0.98^a^	0.45^a^

PPG = preperimetric glaucoma, IOP = intraocular pressure, SE = spherical equivalent, D = diopter, MD = mean deviation, CVA = color visual acuity, logMAR = logarithm of minimal angle resolution, BW = black/white, R = red, GY = green/yellow, BG = blue/green, BP = blue/purple

^a^ Mann-Whitney U test

^b^ Fisher’s test

**Fig 2:**
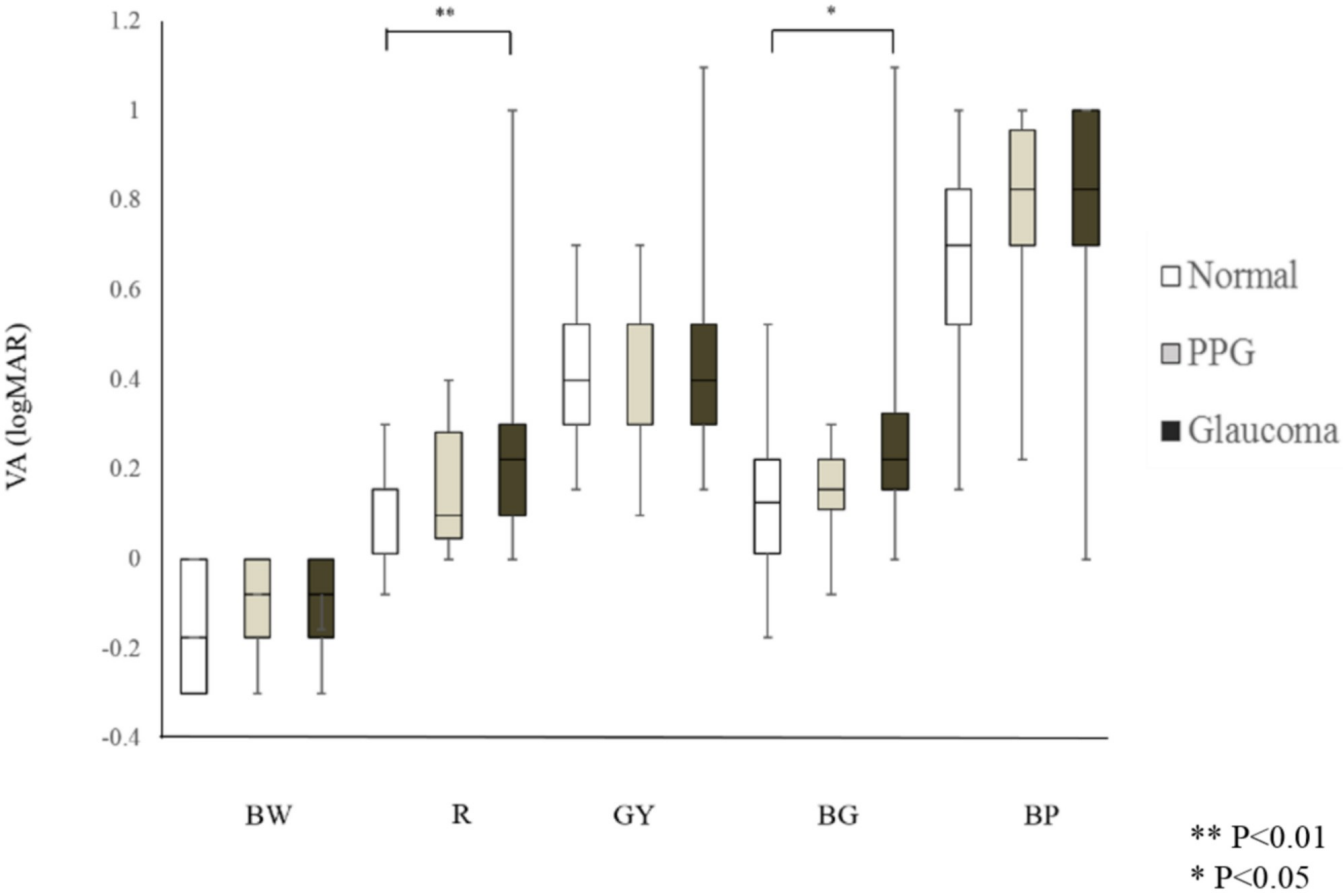
Color visual acuity (VA) for different colors in normal, preperimetric glaucoma (PPG) and glaucoma eyes. Color VA for all colors was similar in the normal and PPG eyes, as well as in the PPG and glaucoma eyes. Red (R) VA and blue-green (BG) VA were significantly lower in the glaucoma eyes than in the normal eyes (*P* = 0.008 and *P* = 0.015, respectively), although black-white (BW) VA, green-yellow (GY) VA and blue-purple (BP) VA were not significantly lower.

CVA for all colors (measured in logMAR) was similar in the normal and PPG eyes, as well as in the PPG and glaucoma eyes (Table 1 and Fig 2). R VA and BG VA were significantly worse in the glaucoma eyes than in the normal eyes (*P* = 0.008 and *P* = 0.015, respectively), although BW VA (i.e., VA measured according to the standard methods), GY VA and BP VA were not significantly worse (*P* = 0.21, *P* = 0.25, and *P* = 0.45, respectively, Table 1 and Fig 2).

**Fig 3:**
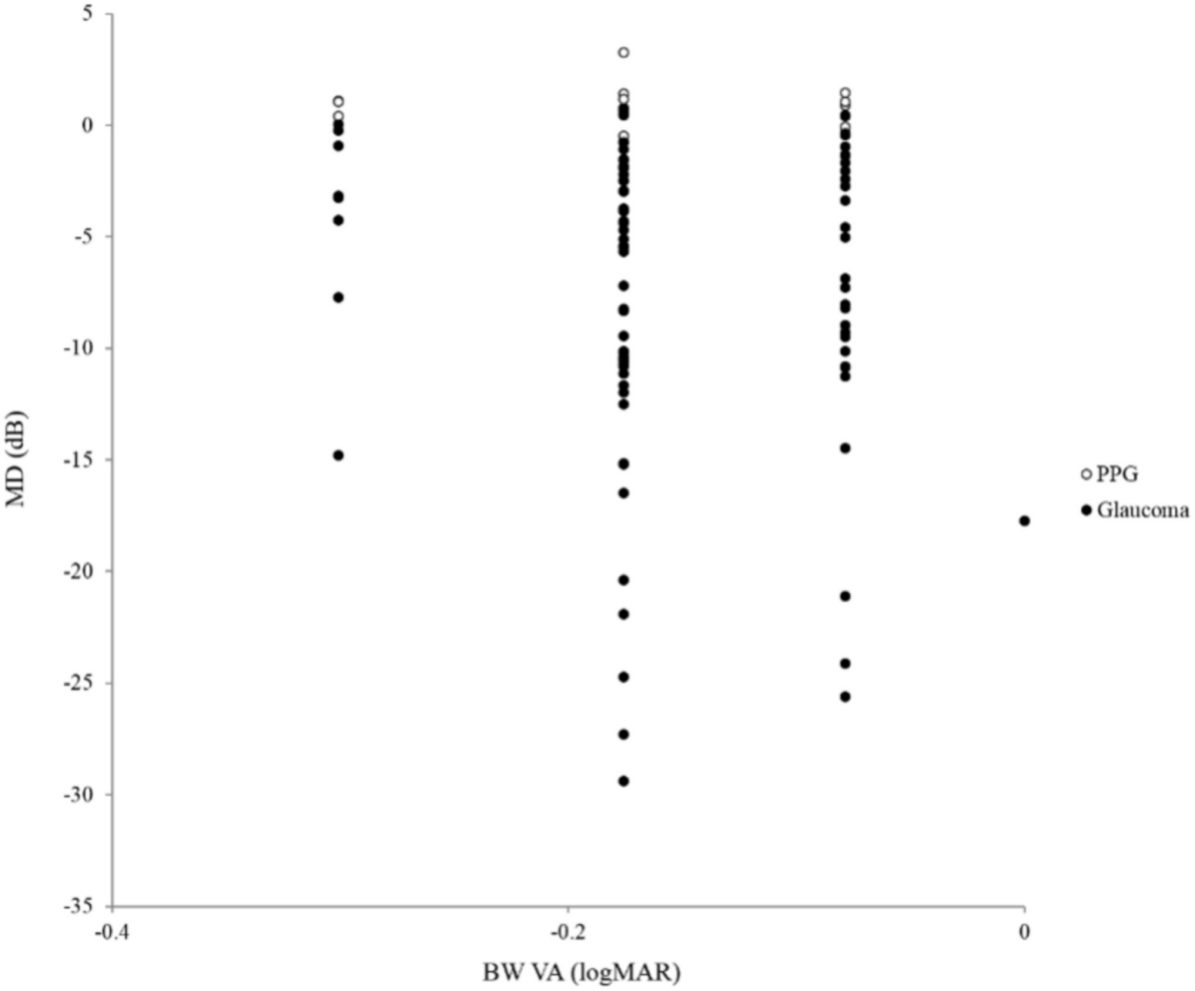
Relationship between mean deviation (MD) and black-white visual acuity (VA) in preperimetric glaucoma (PPG) and glaucoma subjects. Black-white VA was not correlated with MD in the eyes with PPG or glaucoma (r = -0.08, P = 0.45).

**Fig 4:**
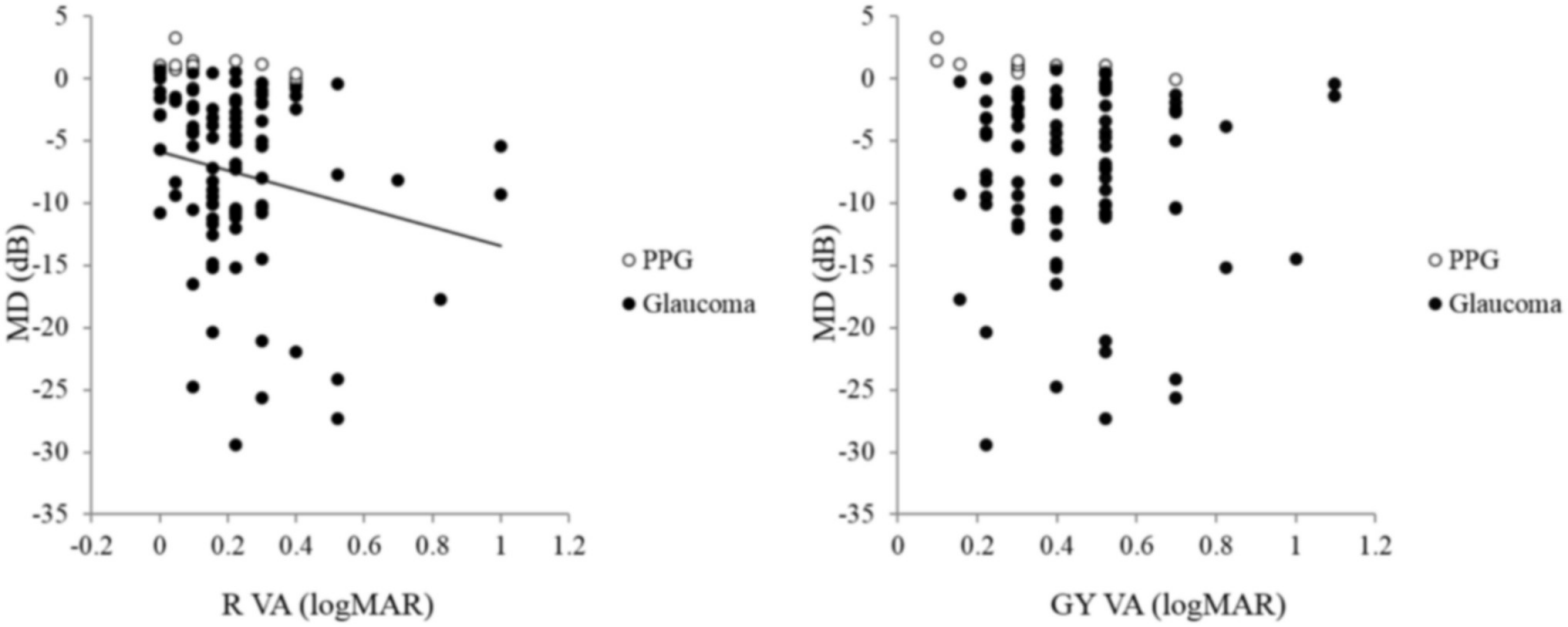
Relationship between mean deviation (MD), red (R) visual acuity (VA) and green-yellow (GY) VA in preperimetric glaucoma (PPG) and glaucoma subjects. R VA was significantly correlated with MD in the eyes with PPG and glaucoma (r = -0.23, *P* = 0.02, left), but GY VA was not (r = -0.09, P = 0.37, right).

**Fig 5:**
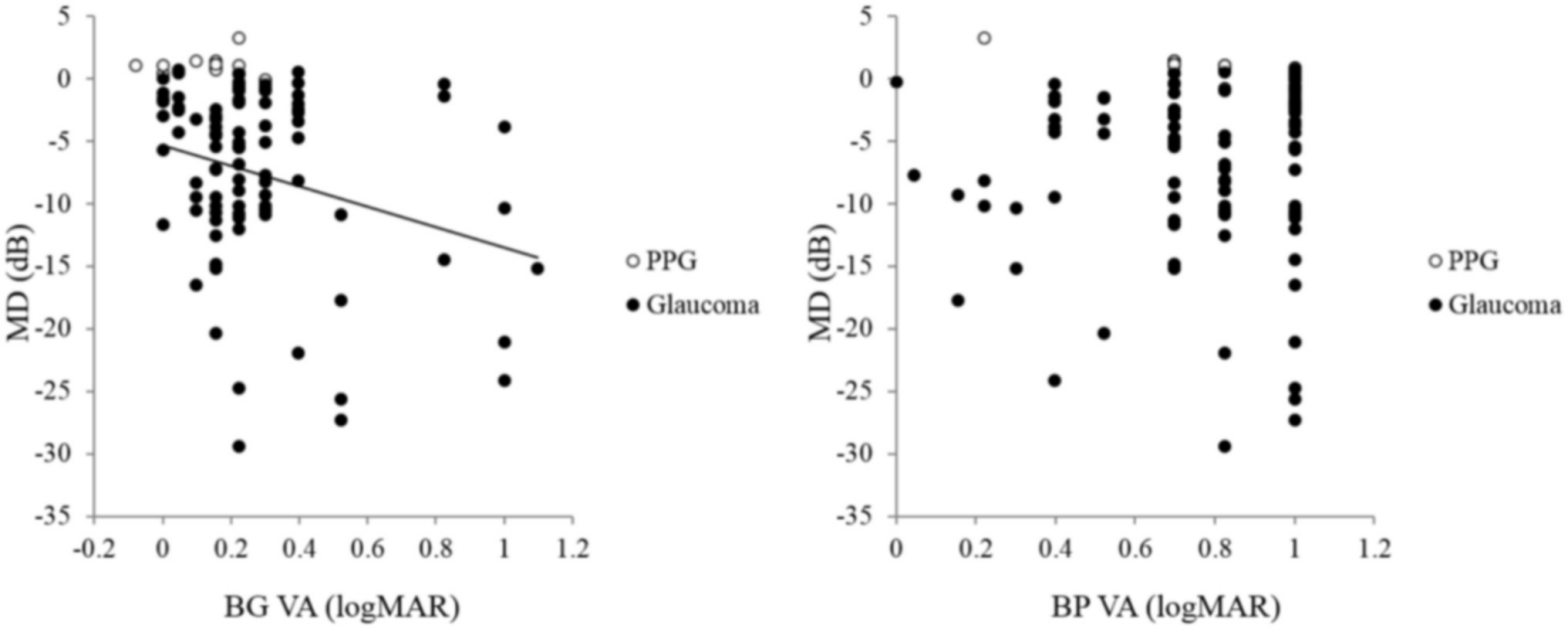
Relationship between mean deviation (MD), blue-green (BG) visual acuity (VA) and blue-purple (BP) VA in preperimetric glaucoma (PPG) and glaucoma subjects. BG VA was significantly correlated with MD in the eyes with PPG and glaucoma (r = -0.25, *P* = 0.01, left), but BP VA was not (r = -0.02, P = 0.87, right).

Furthermore, BW VA was not correlated with MD in the PPG or glaucoma eyes (r = -0.08, *P* = 0.45, Fig 3). R VA and BG VA were significantly correlated with MD in the PPG and glaucoma eyes (r = -0.23, *P* = 0.02, Fig 4 left; r = -0.25, *P* = 0.01 Fig 5 left, respectively), but GY VA and BP VA were not (r = -0.09, *P* = 0.37, Fig 4 right; r = -0.02, *P* = 0.87, Fig 5 right, respectively).

## Discussion

In this study, we tested CVA for R, GY, BG and BP using a paper-based chart with Landolt rings to investigate changes in CVA in PPG and glaucoma patients and healthy controls. Interestingly, we found that R VA and BG VA were detectably impaired in eyes with glaucoma, in close association with the degree of functional loss. Contrarily, CVA for other colors, such as GY VA and BP VA, was not impaired in eyes with glaucoma and was not associated with glaucoma severity. This suggests that measuring CVA with the chart described here may be a promising supplement to existing methods of detecting glaucoma and evaluating its severity.

### New CVA testing system

The most important components of human vision are spatial perception (comprising VA and contrast sensitivity), temporal perception (such as central critical fusion frequency), and color perception. The use of Landolt rings as a stimulus allows color spatial perception to be measured for each color as a logarithmic value (logMAR). Recent improvements in LCD display technology have enabled the development of a testing system to evaluate color visual performance, i.e., CVA.[18, 19] This testing system consists of a personal computer with a color-calibrated, high-accuracy LCD display, allowing the stimulus and background to have equivalent luminance. Older tests, such as the Ishihara Color Test, Standard Pseudoisochromatic Plates and Farnsworth Dichotomous Test Panel D-15, can provide various values, but these values represent fundamentally different properties of vision than color visual acuity, which is what we were interested in here. Furthermore, these tests require a significant amount of time and have historically been limited by using a paper-based approach, which has been relatively limited in its ability to precisely reproduce colors. An initial demonstration of LCD system-measured CVA in normal eyes of younger and older subjects showed the benefits of this system.[18, 19] In the present study, based on previous work, we developed a paper-based color chart to evaluate CVA. We chose this method because of its ease of use; furthermore, patients, as well as ophthalmologists, are generally familiar with VA values, i.e., spatial perception. Furthermore, our chart minimized differences in the chromatic intensity of the stimulus and background.

### Acquired color vision defects and glaucoma

The current study is the first to show that CVA for some colors, such as R VA and BG VA, was detectably lower in eyes with glaucoma than healthy eyes. This finding points to the role of glaucoma as a cause of acquired color vision defects. The main cause of glaucoma is elevated IOP, which can also affect the cone cells.[21–24] Cone cells are classified by their pigment into three types: L-, M-, and S-cones. The L-cones are the most common type (more than 50%), followed by the M-cones, with the S-cones comprising only a very minor proportion (less than 10%) of the cone cells.[25, 26] Furthermore, the S-cones are highly susceptible to damage,[27] which might lead to acquired blue/yellow color vision deficiency, though this remains the subject of debate. Thus, color vision deficiency due to S-cone impairment occurs before deficiency due to L- and M-cone impairment in glaucoma as well as retinal disease. However, the L- and M-cones, which are mainly associated with red and green perception, respectively, have been reported to be preferentially affected in experimental models of glaucoma.[28] Thus, since R VA and BG VA are associated with the L- and M-cones, respectively, we consider that our current results represent a confirmation of these previous results. It is also historically well known that blue-on-yellow perimetry can detect S-cone impairment at an earlier stage of glaucoma.[29] In comparison with standard white-on-white perimetry, blue-on-yellow perimetry is known as an early indicator of glaucomatous damage,[30] and has been reported to be effective in predicting which patients with early glaucomatous visual field loss are most likely to continue to progress.[31] Nevertheless, significant differences in GY VA and BP VA, which are associated with the S-cones, were not present in the PPG and glaucoma patients here. This might suggest that S-cones do not mediate visual acuity, perhaps due to their low distribution in the macular region. Furthermore, we cannot compare the results of perimetric testing with CVA, which was used in this study, because CVA measures foveal color visual function as the minimum angle of resolution. Thus, the two techniques return fundamentally different results. Further investigation is needed, but the number of S-cones in the macular region is normally far lower than other cone types, and correspondingly, GY VA and BP VA are relatively low even in healthy subjects.[25] Therefore, it is reasonable that we could not detect impairment in GY VA or BP VA in our PPG and glaucoma patients. This might also explain our results that CVA for any color was not lower in eyes with PPG than healthy eyes, though the exact reason still remains unclear.

Interestingly, the current study is the first to show that R VA and BG VA were associated with MD in patients with glaucoma. This result was due to our inclusion of patients with PPG in the analysis. This inclusion reflects the current view that there is a “glaucoma continuum,[32, 33] with structural changes beginning in PPG and progressing continuously until the last stages of the disease. Thus, our findings on R VA and BG VA suggest that impairment of the M- and L-cones in the macula is associated with glaucoma severity in the peripheral retina, and continues in parallel with glaucoma progression. However, efforts to develop structural analytical methods of estimating baseline RGC density based on cone density may offer little advantage over simple measurements of RGC density in identifying early glaucomatous change.[34] Even if cone density remains normal, impairment of the connected RGCs will prevent the cones from functioning properly, and CVA in the associated location can be expected to worsen. Thus, we consider that the new test of visual function described here may be an important complement to structural measurement techniques in the detection of RGC loss.

### Glaucoma progression

The development of simple, easy-to-use methods of detecting glaucoma and monitoring its progression is an important goal of glaucoma research. Approaches to achieve this goal include structural, functional, and blood flow analyses. Blue-on-yellow perimetry has been reported to be an early indicator of glaucoma,[30, 31] and frequency doubling technology perimetry has also been reported to be effective in detecting early glaucomatous losses.[35, 36] It is also well known that OCT measurements of cpRNFLT can identify the presence of glaucoma, and that cpRNFLT decreases with the severity of glaucoma.[37–42] Several studies of glaucoma have also evaluated the diagnostic performance of measurements of macular ganglion cell complex thickness, and have reported that their accuracy is similar to measurements of cpRNFLT, even in highly myopic eyes.[43–45] OCT and new perimetric techniques have also allowed sensitive detection of glaucomatous morphological changes and visual field impairment, thereby enabling assessment of disease progression.[46–49] Other research has focused on ONH blood flow impairment, which has been suggested to be involved in glaucoma progression.[50–53] In the current study, there was in fact significant overlap between two of the hues, i.e., R and BG, making them unsuited for the clinical diagnosis of glaucoma. Thus, CVA measurement with the new visual color function test described here is most promising as a complement to recent technological advancements in detecting glaucoma and evaluating its severity.

### Limitations

Our study had several limitations. First, it was a cross-sectional study enrolling only Japanese subjects, including the healthy controls, PPG patients, and glaucoma patients. To avoid IOP-related biases, the glaucoma subjects included only patients with IOP-controlled OAG, and the PPG subjects excluded patients with ocular hypertension. This decision was made because the effect of high IOP on CVA remains unclear. The glaucoma patients were carefully selected by an experienced glaucoma specialist (T.N.). In fact, the PPG group included 7 out of 14 eyes with MD greater than 1.0 dB, while the normal group included only 2 such eyes out of 22. This, combined with the small number of PPG eyes, may explain the inverse difference between normal and PPG subjects in MD. Second, though all patients had best-corrected VA ≥ 20/20 and no patients had central visual field defects, we included patients with all stages of OAG. Thus, the OAG patients overall had relatively severe glaucoma, with a mean MD of about -8 dB. Despite this, a significant number of the OAG patients had relatively normal CVA, suggesting that future investigation of the precise relationship between MD and CVA might be valuable. Furthermore, some of the older individuals in the study are likely to have had cataract. Even if this did not significantly affect their BCVA (all patients had BCVA 20/20) this may have been a confounding factor in the CVA results. Third, though we produced a paper-based color chart that minimized differences between the chromatic intensity of the stimulus and the background (i.e., isolating the difference to hue), there might have been some difference in intensity between the stimulus and background for the GY rings, perhaps caused by limitations of current printers. This might explain our finding that GY VA was better in all subject groups than BP VA. In principal, GY and BP VA both reflect the condition of the S-cones, and these two types of CVA should therefore be equivalent. Fourth, M- to L-quantum catches may support spatial discrimination, possibly leading to red-green color discrimination being correlated with BCVA to some degree. However, we did not obtain any results to support such an association. This might be explained by the fact that the sampling unit for color acuity is by necessity larger than for achromatic contrast, and that the color acuity test in effect assays more peripheral cells. Fifth, it is difficult to balance the color of the chart (D60) under all possible clinical light conditions, even with advanced devices, such as the Minolta CS 2000. However, we have included information on the theoretical chromaticity of the chart, and the results of Konica-Minolta FD7 measurements, to Fig 1 and S1 Table. These data show that there were some gaps between the theoretical and measured values for the GY and BP colors. However, these were minor and should not have affected the results.

### Conclusions

In conclusion, this study investigated changes in CVA in PPG and glaucoma patients using a paper-based chart with Landolt’s rings. We found that R VA and BG VA were detectably impaired in eyes with glaucoma, in close association with the degree of functional loss. Contrarily, CVA for GY VA and BP VA was not impaired in eyes with glaucoma, and there was no association with glaucoma severity. Our findings suggest that measuring various types of CVA with the new color vision test described here may be a promising supplement to existing methods of detecting glaucoma and evaluating its severity.

## Supporting information

S1 Table Characteristics of chromaticity, delta and Michelson contrast. (PPTX)

S2 Table All the raw data in the study. (XLSX)
