# Supplementary material for: Color visual acuity in preperimetric glaucoma and open-angle glaucoma
Source: PLoS One. 2019 Apr 17;14(4):e0215290. doi: 10.1371/journal.pone.0215290 (PMC6469804; doi:10.1371/journal.pone.0215290)
Supplement: S1 Table — (PPTX) [file pone.0215290.s001.pptx]

## Slide 1
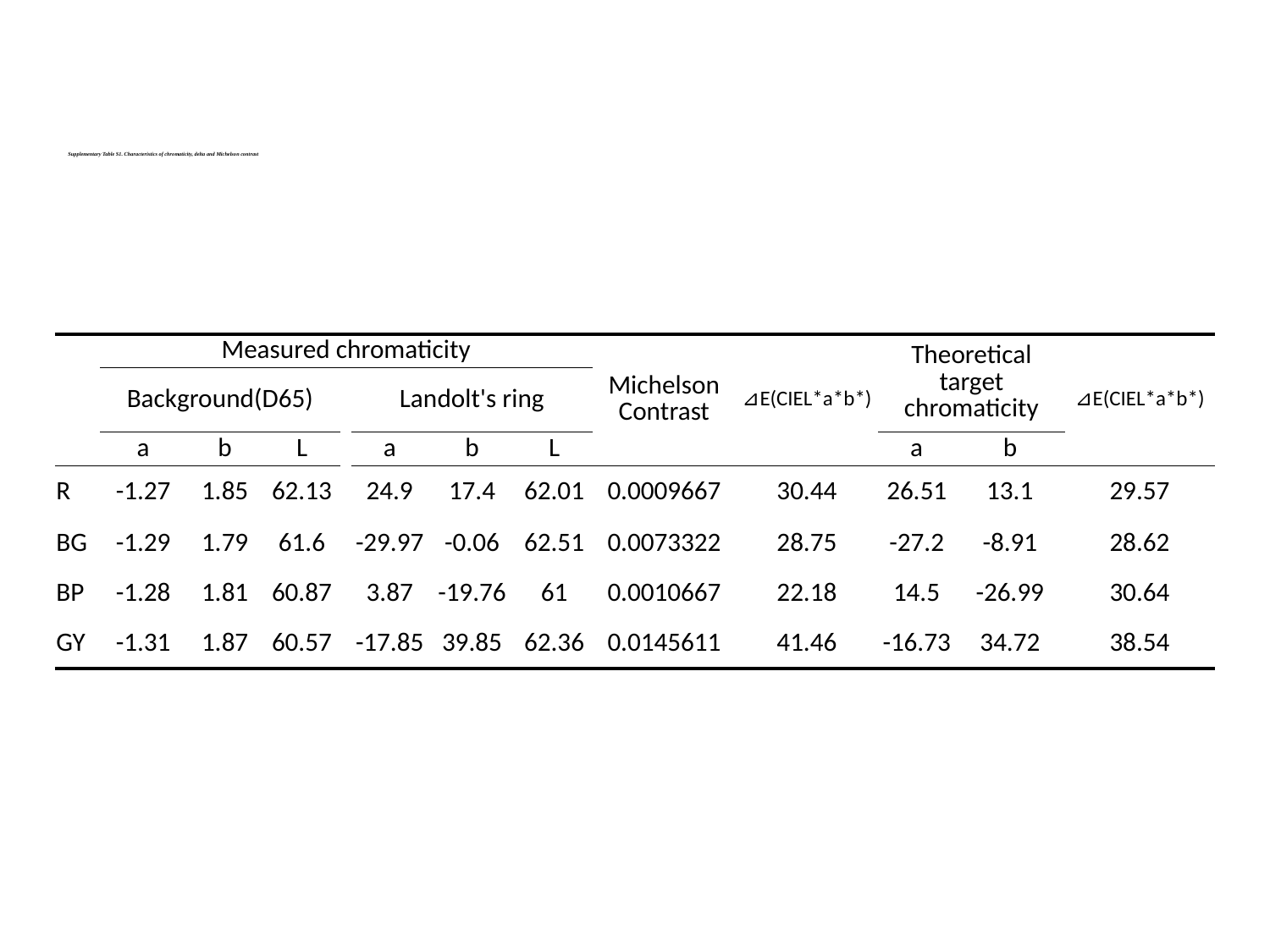

# Supplementary Table S1. Characteristics of chromaticity, delta and Michelson contrast
| | | | | | | | | | | | | | | | | | | | |
| --- | --- | --- | --- | --- | --- | --- | --- | --- | --- | --- | --- | --- | --- | --- | --- | --- | --- | --- | --- |
| | Measured chromaticity | | | | | | | | | | | | | Michelson Contrast | ⊿E(CIEL\*a\*b\*) | Theoretical target chromaticity | | | ⊿E(CIEL\*a\*b\*) |
| | Background(D65) | | | | | | | Landolt's ring | | | | | | | | | | | |
| | a | | b | | L | | | a | | b | | L | | | | a | b | | |
| R | -1.27 | | 1.85 | | 62.13 | | | 24.9 | | 17.4 | | 62.01 | | 0.0009667 | 30.44 | 26.51 | 13.1 | | 29.57 |
| BG | -1.29 | | 1.79 | | 61.6 | | | -29.97 | | -0.06 | | 62.51 | | 0.0073322 | 28.75 | -27.2 | -8.91 | | 28.62 |
| BP | -1.28 | | 1.81 | | 60.87 | | | 3.87 | | -19.76 | | 61 | | 0.0010667 | 22.18 | 14.5 | -26.99 | | 30.64 |
| GY | -1.31 | | 1.87 | | 60.57 | | | -17.85 | | 39.85 | | 62.36 | | 0.0145611 | 41.46 | -16.73 | 34.72 | | 38.54 |
| | | | | | | | | | | | | | | | | | | | |
